# The Neutrophil’s Choice: Phagocytose vs Make Neutrophil Extracellular Traps

**DOI:** 10.3389/fimmu.2018.00288

**Published:** 2018-02-20

**Authors:** Angelo A. Manfredi, Giuseppe A. Ramirez, Patrizia Rovere-Querini, Norma Maugeri

**Affiliations:** ^1^Università Vita-Salute San Raffaele, Milano, Italy; ^2^Division of Immunology, Transplantation and Infectious Diseases, IRCCS Ospedale San Raffaele, Milano, Italy

**Keywords:** neutrophils, apoptosis, phagocytosis, NETs, platelets

## Abstract

Neutrophils recognize particulate substrates of microbial or endogenous origin and react by sequestering the cargo *via* phagocytosis or by releasing neutrophil extracellular traps (NETs) outside the cell, thus modifying and alerting the environment and bystander leukocytes. The signals that determine the choice between phagocytosis and the generation of NETs are still poorly characterized. Neutrophils that had phagocytosed bulky particulate substrates, such as apoptotic cells and activated platelets, appear to be “poised” in an unresponsive state. Environmental conditions, the metabolic, adhesive and activation state of the phagocyte, and the size of and signals associated with the tethered phagocytic cargo influence the choice of the neutrophils, prompting either phagocytic clearance or the generation of NETs. The choice is dichotomic and apparently irreversible. Defects in phagocytosis may foster the intravascular generation of NETs, thus promoting vascular inflammation and morbidities associated with diseases characterized by defective phagocytic clearance, such as systemic lupus erythematosus. There is a strong potential for novel treatments based on new knowledge of the events determining the inflammatory and pro-thrombotic function of inflammatory leukocytes.

## Neutrophils Recognizing Particulate Substrates

Neutrophils activate stereotyped programs when the environment changes. As most professional phagocytes, they react when challenged with bulky preys. Neutrophils kill microbes *via* phagocytosis, generation of oxidant species, and activation of the cell proteolytic machinery, processes that have been extensively studied in the last decades. The release of neutrophil extracellular traps (NETs) vicariates frustrated or ineffective phagocytosis. It enhances the efficacy of the innate response coping with invading microbes. Moreover, NETs counterbalance microbial strategies to evade the immune response. NETs are large macromolecular structures that comprise neutrophil DNA, citrullinated histones, and an array of active proteases (1, 2). Because of the adhesive properties of nucleic acids and of the action in the extracellular environment of histones and of neutrophil enzymes, NETs contribute to the host defense against various microbial species (3). They form a three-dimensional template absorbing and retaining players of the humoral innate immune response, the prototypic long pentraxin, pentraxin 3, and complement (4–8).

Excellent recent reviews detail the mechanisms involved in the generation of NETs and we remand to them interested readers (3, 9–13). Of importance, NET formation and extrusion implies a dramatic rearrangement of the neutrophil intracellular architecture. Chromatin decondensation is a prerequisite for NET assembly and depends on the citrullination of histones driven by the PAD4 enzyme (14, 15), by the action of DEK, a nuclear chromatin protein involved in epigenetic and transcriptional regulation (16), and by the concerted action of enzymes, which are partially complexed in the azurophilic primary granules, myeloperoxidase, and elastase. Elastase action is responsible of the partial proteolytic processing necessary to disrupt chromatin packaging (17–19). von Willebrand factor adsorbed to NETs, citrullinated histones, and nucleic acid negative charges concur to the recruitment and the activation of platelets, thus impacting on hemostasis and eventually favoring thrombosis. Thrombosis initiated by the innate immunity, also referred to as “immunothrombosis,” plays an increasingly recognized role in vessel protection, limiting the intravascular growth and the hematogenic spread of infectious agents (5, 20–23). Conversely, endogenous mechanisms involving DNase1 and DNase1-like 3 control the thrombogenic potential of NETs *in vivo*, under conditions where microbial and sterile stimuli are responsible for the activation of neutrophils (24).

In this essay, we focus on neutrophils that face non-microbial “unconventional” particulate substrates, apoptotic cells, and activated platelets in particular. Non-infectious particulates do not usually pose a direct challenge to the integrity of the organism. However, they cause neutrophil responses surprisingly similar to those caused by microbes, including most notably phagocytosis and generation of NETs. Neutrophils avidly phagocytose apoptotic cells and are the prominent scavengers of cell remnants in biological fluids, the blood in particular, where they represent the counterpart of scavenger macrophages in solid tissues. Neutrophils that had internalized extracellular nuclei are referred to as “LE cells,” and they represent a virtually unique feature of the prototypical systemic autoimmune disease, systemic lupus erythematosus (SLE) (25, 26). Initially, the LE phenomenon was thought to reflect the lysis of a neutrophil lobe. In contrast, the intracellular vesicles contain entire nuclei that had actually been phagocytosed, transferred into the phagolysomes, and partially digested. Phagocytosis has been confirmed by flow cytometry (27, 28) and depends on factors in the biological fluids of patients with SLE, in particular autoantibodies recognizing nuclear antigens, histones and DNA, and complement (25). LE cells have been originally identified in the bone marrow of lupus patients. They have been found in the blood, synovial and cerebrospinal liquids, and serosal effusion (25, 26). The LE phenomenon can be induced *in vitro* and, besides highlighting the importance of opsonizing signals, reveals that whole nuclei are frequently present in biological fluids. This might depend on pyroptosis, an inflammatory form of cell death, in which entire nuclei surrounded by the nuclear membrane are released together with inflammatory cytokines in the microenvironment (29).

Indeed cytokines and other signals important in phagocyte biology, including the growth factor, GM-CSF, are known to enhance the ability of neutrophils in the fluid phase to recognize and to internalize apoptotic cells (30–32). Conversely, the depletion of phagocytes before sterile acute tissue injuries causes the accumulation of cell debris, influencing the outcome of the repair and the associated immune response (33, 34). Thus, neutrophils emerge as key players in the maintenance of tissue homeostasis in physiological conditions (35).

Neutrophils and platelets frequently and extensively interact in the peripheral blood and at sites of inflammation. Their cross-talk is important in the maintenance of the homeostasis. Platelets scan the vascular surface and collect deposited bacteria, boosting neutrophil activities such as NET generation (36), while the deregulated neutrophil–platelets interaction plays a role in the pathogenesis of rheumatic diseases (21, 37, 38) and severe sepsis (39). Platelets and neutrophils adhere and form heterotypic aggregates, which are found not only in the blood of patients with inflammatory or autoimmune diseases but also in the blood of patients with cancer and acute coronary syndromes (40). Heterotypic aggregates sustain and amplify the activation of platelets and neutrophils, fostering their inflammatory, antibacterial, hemostatic, and pro-thrombotic actions (11). The ability to release cytokines, chemokines, and vasoactive molecules and the enhanced ability of leukocytes to extravasate and to reach inflamed tissues reflect the reciprocal activation of platelets and neutrophils (36, 41–44).

Neutrophil–platelet initial interaction depends *in vitro* and *in vivo* on platelet P-selectin interaction with the PSGL1 receptor on leukocytes (45–49). Because of the initial tethering event, neutrophils redistribute their vesicular content and expose on the plasma membrane biologically active molecules, such as myeloperoxidase and tissue factor, which are normally contained in the granules or in the cytoplasm. Moreover, they upregulate the expression of phagocyte β2 integrins that are transactivated, acquiring a higher affinity for the fibrinogen presented by platelet α integrins. The latter interaction stabilizes the adhesion between the phagocyte and the platelet (50–52) (Table 1).

**Table 1:** Some defined platelet/neutrophil molecular interactions.

Platelet/platelet-derived microparticles	Bridging moiety	Neutrophil	Possible outcome	Main relevant references
P-selectin	None described	PSGL1	Neutrophil β2 integrin upregulation/transactivation	(50, 53, 54)
			Neutrophil degranulation	(51, 55–57)
			ROS generation	(58, 59)

PS	*Gas-6?*	*MERTK?*	Platelets clearance *via* phagocytosis	(51)
	*Protein S?*	*AXL?*		
	*MFG-8*	*RAGE?*	Phagocyte hyporesponsiveness to further inflammatory stimuli	(51)
	*Others?*			

HMGB1	None described	RAGE	Neutrophil β2 integrin upregulation/transactivation	(58, 60, 61)
			Pericellular distribution of myeloperoxidase and elastase from primary granules	(58, 60, 61)
			Mitochondrial ROS formation	(23, 62, 63)
			Autophagy	(58, 61)
			Inflammatory-mediated tissue damage	(60, 64, 65)
			NETs generation and thrombosis	(58, 65)

Glycoprotein Ib		Activated Mac-1	Adhesion of resting platelets to activated neutrophils	(63)

αIIβ3	Fibrinogen	Activated Mac-1	Adhesion of resting platelets to activated neutrophils	(66)

*MFG-E8, milk fat globule-EGF factor 8; NET, neutrophil extracellular trap; ROS, reactive oxygen species*.

Depending on environmental conditions, the phagocyte metabolism, activation and interaction with the extracellular matrix, and still poorly identified signals expressed/released by the tethered platelets, three outcomes can be envisaged. First, at the end of the sustained interaction, leukocytes dissociate from platelets—possibly because of active proteolysis of ligands by neutrophil enzymes—and reach the inflammatory sites where they exert their effector function (41–43, 55, 67).

Second, active engulfment takes place, which is exquisitely dependent on the recognition of a common feature of activated platelets, i.e., the exposure of anionic phospholipids, such as phosphatidylserine (PS). The direct or indirect recognition of PS on the prey can result in the rearrangement of actin-based cytoskeleton and the internalization of the tethered platelet by professional and non-professional phagocytes, such as endothelial cells (51, 55, 68, 69). Under physiological conditions, recognition and phagocytic clearance of activated platelets purge the bloodstream of procoagulant stimuli while quenching the neutrophil sensitivity to inflammatory stimuli (see below) (70). The phagocytic removal of activated platelets conforms to the “tether and tickle” model originally proposed for the removal of apoptotic dying cells by Fadok, Henson, and collaborators (71, 72). This is a two-step model in which (i) the dying cell is initially tethered to the phagocyte and (ii) other interactions based on the direct or indirect recognition of PS transduce signals that initiate cell internalization (73, 74). In the case of activated platelets, the initial tethering depends on P-selectin recognition and immediately downstream events, while the availability of PS appears crucial for actual internalization of the phagocytic substrate (51, 55, 68, 69).

Third, the firm interaction between neutrophils and tethered PS-expressing phagocytic substrates is a potent inducer of NETs (see below). Indeed, regulation of the overall charge of the phagocytic substrate, like it occurs during apoptosis when glycosylated epitopes undergo caspase-dependent desialylation, influences the interaction between phagocyte and prey and the efficacy of the clearance (75).

## PS and Cell Clearance Programs

Phospholipid translocases, scramblases, and flippases, maintain the asymmetry of the plasma membrane phospholipids. Upon platelet activation triggered by various agonists, the intracellular Ca^2+^ concentration sharply increases, interfering with the translocase action and causing the rapid, patchy, potentially transient exposure of PS (76). The pathway involves the disruption of the platelet inner mitochondrial membrane, an event underlying PS exposure by activated, apoptotic and senescent platelets (77). Activated cells often expose PS without being phagocytosed. This points out to the existence of “do not eat me” signals. The dynamics of exposure of PS represents another variable (78). PS recognition leads to phagocytosis only when PS aggregation by tethering receptors causes firm and lasting interactions between the phagocyte and the prey. Such tethering receptors comprise Tim4 for apoptotic cells and possibly PSGL1 for activated platelets (78, 79). Finally, cells dying because of caspase-mediated programmed cell death might expose modified PS residues, thus providing a better substrate for recognition from at least some PS receptors (see below) and tagging cells for phagocytic clearance (80, 81).

In support, hindrance with the recognition of oxidized lipids interferes with the interaction between phagocyte and prey and the ensuing clearance of apoptotic cells (82). Moreover internalized oxidized phospholipids and oxysterols cause the activation of PPAR-delta receptors (83) and the LXR nuclear receptor in macrophages (84) in turn inducing the expression in macrophages of signals that further enhance the process such as the Mer receptor (84).

Oxidation-specific epitopes in general are recognized by various pattern recognition receptors and components of the humoral innate immune systems, tagging for removal damaged cells and low-density lipoproteins. Of interest, natural IgM antibodies specifically and effectively bind to oxidized epitopes on blood microparticles, quenching their ability to trigger the production of inflammatory signals, IL-8 in particular from macrophages (85). Conversely, the accumulation of oxidized moieties *per se* cause unrelenting inflammation and contribute to various human vascular disease, including atherosclerosis (85–87).

Diverse receptors recognize PS, either directly or through the moieties that PS binds on the outer leaflet of the plasma membrane. Receptors include Tim4, the tyrosine kinase receptors Tyro3, Axl, and Mer (78, 88, 89). “Bridging” molecules comprise structurally and functionally heterogeneous soluble ligands such as Protein S, Gas-6, and milk fat globule-EGF factor 8 (MFG-E8). Microparticles released from activated platelets bind *via* PS to Protein S and Gas-6 (90). Gas-6 stabilizes the interaction among activated platelets, endothelial cells, and leukocytes facilitating heterotypic cell aggregation in the blood (91), while the interaction among PS, GAS-6, and the Axl receptor mediates microparticle clearance (90). MFG-E8 has been as well implicated in the formation of heterotypic aggregated and in PS-mediated clearance of platelets in *in vivo* models of sepsis (69). Human neutrophils constitutively express the Mer receptor and upregulate its expression in septic conditions (92) (Table 1). Other molecules related to the humoral innate immune responses such as the beta2-glycoprotein 1, pentraxins, and complement fractions bind to apoptotic cells and activated platelets influencing their immunogenicity either because they provide a template for autoantibody binding or because they facilitate the local generation of adjuvant signals (8, 93–100).

Direct or indirect PS recognition is required on the one hand for the efficacy of phagocytosis. It also causes the selective production of cytokines such as IL-10 and TGF-β and specialized classes of pro-resolving lipidic mediators such as resolvins (101–106) that prompt the active termination of the inflammatory response. In concert with the cytokines, IL-4 and IL-13, PS recognition links tissue damage to tissue repair by the reprogramming of local macrophages (107), which in turn guide the activation, the proliferation, and the survival of stem and progenitor cells (108–111).

Tampering with the recognition of PS or PS-associated moieties thus leads to the accumulation of apoptotic debris, to persisting unrelenting inflammation, and to the failure in the ability of injured or damaged tissues to heal. Moreover, it is closely associated with the development of autoimmunity, often with serological and clinical features of the prototypic systemic autoimmune disease, SLE (86, 112–116). Autoimmunity follows the cross-presentation of apoptotic cell antigens to autoreactive T cells in genetically susceptible backgrounds (114, 117–125). Autoimmunity, unrelenting inflammation, and accumulation of cell remnants associate in human SLE and in most SLE experimental models with alteration of blood neutrophils (126). Exogenous MFG-E8 corrected *in vivo* most alterations (see above), highlighting the link of systemic autoimmunity, defective neutrophil function, and the recognition of PS (127).

## When the Game is Tough, Neutrophils Look for Company

As discussed above, the interaction with particulate substrates can trigger the production of NETs (128). The seminal work of Clark and collaborators has revealed that in experimental models of sepsis, bacterial LPS primarily activate the TLR4 of platelets. In turn, TLR4-activated platelets interact with neutrophils and commit them to the generation of NETs NETs (39, 129). Besides microbial constituents, sterile stimuli leading to platelet activation cause the generation of signals that trigger neutrophil activation and favor the production of NETs (58). HMGB1 is a prototypic endogenous inflammatory signal, which is expressed by platelets, is released upon activation, and represents a master regulator of leukocyte inflammatory activation and thromboinflammation (60–62, 130–135). Indeed the presentation of bioactive HMGB1—either soluble or associated with the plasma membrane of tethered platelets or of platelet-derived microparticles (58, 60–62, 64, 65, 130)—to neutrophils represents a non-redundant signal, by which platelets instruct neutrophils to release NETs, *via* a pathway that involves the HMGB1 receptor expressed by neutrophils, RAGE (13). Platelets represent a “barometer” used by neutrophils to decide whether they should undergo activation. NETs generation occurs when inflammatory stimuli of microbial or endogenous origin exceed a threshold acceptable for platelets only (11, 39, 49).

## Neutrophils That have Phagocytosed Keep Calm

**Figure 1:**
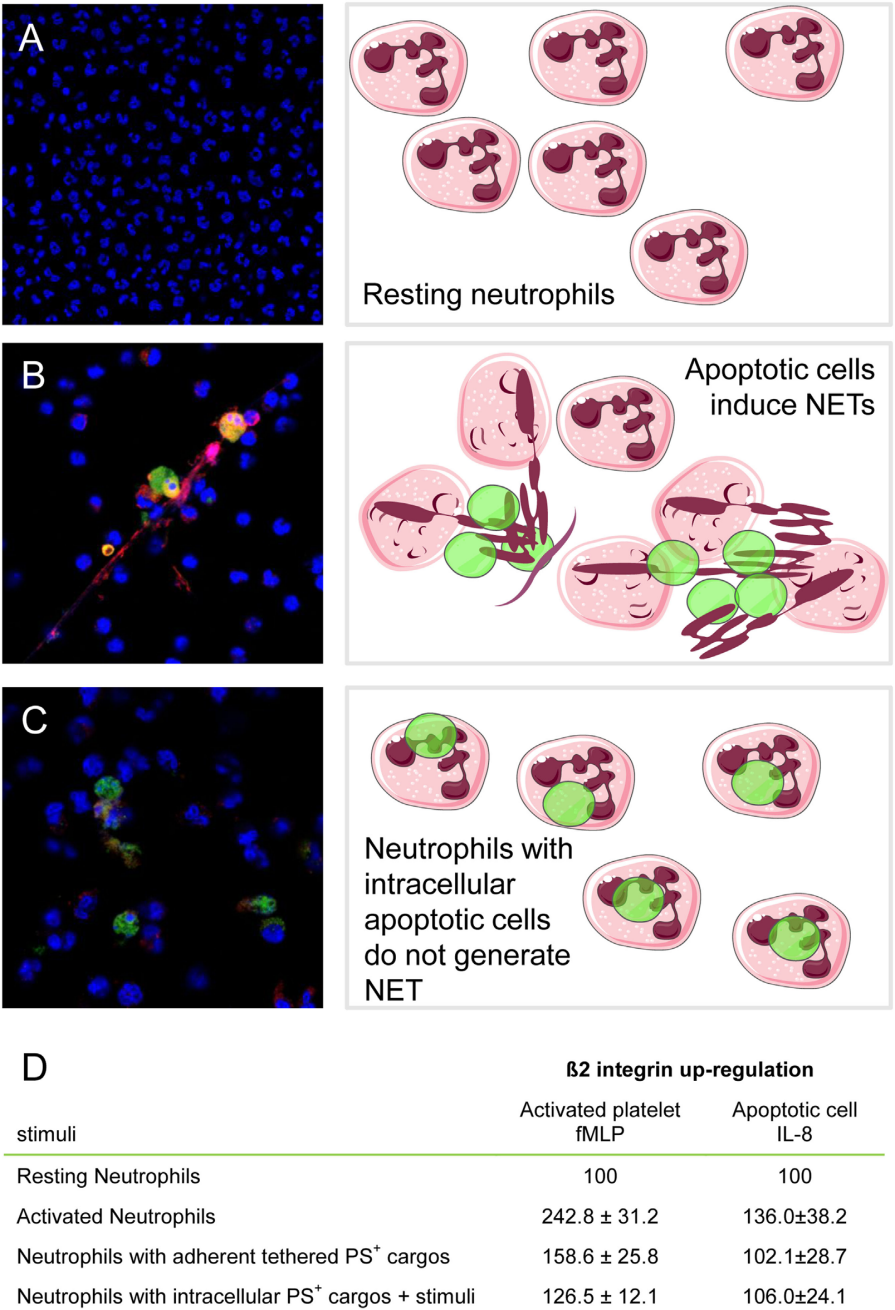
Neutrophils that had phagocytosed apoptotic cells or activated platelets fail to be activated when challenged with further inflammatory stimuli. Neutrophil extracellular traps (NETs) formation was monitored by confocal microscopy. Cathepsin G was revealed by immunofluorescence (Alexa Fluor 541, red), and DNA was counterstained with Hoechst and apoptotic cells preloaded with CFSE (equivalent to Alexa 488, green). **(A)** Unstimulated neutrophils; **(B)** neutrophils challenged with apoptotic cells; **(C)** neutrophils that had phagocytosed apoptotic cells and then were after adherence further stimulated with recombinant IL-8. **(D)** β2 integrins were determined by flow cytometry in resting neutrophils (basal value), neutrophils with adherent tethered PS + cargos (platelets or apoptotic LCL cells), or neutrophils with intracellular PS + cargos (platelets or apoptotic LCL cells) after further stimulation with fMLP or IL-8. Results (mean ± SEM) are expressed as the percentage of basal value. Adapted from Ref. (51, 128).

The platelet barometer works not only by soliciting neutrophil responses but also by switching them off. Neutrophils that had phagocytosed bulky particulate substrates, such as apoptotic cells and activated platelets, appear to be “poised” in an unresponsive state, since they become unable to respond to further inflammatory stimuli and fail to release their granular content or to generate NETs (51, 68, 128, 136). The “calming touch” associated with the phagocytosis of large PS-exposing particulate substrate (137) (Figure 1) might limit the collateral damages to inflamed vessels and tissues by the unrestrained activation of neutrophils (138).

## The Choice of Neutrophils In Front of Crystals and Microparticles

Monosodium urate crystals, whose precipitation is the key event in the pathogenesis of gout (139), are potent NET inducers. Recent elegant studies have shed light on the clinical pattern of early gouty arthritis. This stage of the disease is characterized by acute and transient inflammatory responses to crystals that cannot be eliminated through phagocytosis and persist in the tissues of patients for long periods (140). Early phases after crystal precipitation are characterized by the production of inflammatory cytokines, IL-8 and IL-1β in particular (139, 141), and by the noxious effects of isolated, pro-inflammatory NETs (142). Later neutrophils accumulate within the tissue. The concentration of locally produced NETs increases, favoring NET aggregation. Aggregated NETs provide a proteolytic template, which traps cytokines and chemokines that undergo degradation by the neutrophil proteases associated with the NET DNA backbone. Inactivation of the chemokines and cytokines leads the swift termination of the inflammatory response despite persistence of precipitated crystals (140, 143), while aggregated NETs contribute to tissue damage and remodeling in late phases of the disease, referred to as *tophaceous gout* (142).

Environmental signals and intracellular events shape the transient response elicited by the crystals. They include (i) still uncharacterized signals activating the P2Y6 purinergic receptor/store-operated calcium entry/IL-8 axis (144) and (ii) the presence of IL-1β, combined with the ability of neutrophils to activate the autophagic machinery (145). IL-1β and autophagy-dependent NET generation also play a critical role in Familial Mediterranean fever, possibly the best characterized autoinflammatory disease, whose clinical manifestations comprise transient self-limiting inflammatory phases with fever, polyserositis, and acute phase responses (146). Neutrophil ability to activate autophagy is selectively downregulated during remitting phases of Familial Mediterranean fever *via* upregulation of the stress-related protein REDD1 (regulated in development and DNA damage responses 1) signal, suggesting that remission might be associated with a block of the ability to activate autophagy and to release inflammatory NETs (147).

IL-1β generated *via* macrophage inflammasomes plays a critical role in atherosclerosis, and cholesterol crystals prompt *in vivo* NET generation. Macrophages exposed to NETs respond by activating the TH17 cell-dependent pathway that amplifies and sustains the recruitment of neutrophils within the atherosclerotic plaque (148). Similar self-sustaining positive feedback forward loops might be involved in the establishment and growth of lesions and in the atherothrombotic complications associated with diseases in which neutrophil activation is involved (38, 44, 56, 58, 149–154).

Relatively small (less than 1 μ) urate aggregates are a normal finding in the fluids of patients with hyperuricemia and only a fraction of these subjects develops acute gouty arthritis. Microaggregate clearance by blood neutrophils and monocytes prevents the actual precipitation of crystals, their frustrated phagocytosis, and the generation of NETs (155). Phagocytosis is assisted by fractions of the complement cascade and by the negative acute phase protein, fetuin (155). Therefore, even in the case of crystal recognition, phagocytosis and NET generation represent alternative outcomes of neutrophil activation, which may eventually adjust negatively to each other, as it has been demonstrated extensively for microbial structures [e.g., see Ref. (156)].

Microparticles also trigger NETs generation, playing a role in the pathogenesis of diseases characterized by extensive vascular damage, such as lupus nephritis, systemic sclerosis, and preeclampsia (60, 157–160). Microparticles share signals with activated platelets and apoptotic cells, from which they often originate. Microparticle recognition involves a similar group of receptor/ligand pairs, including the direct or indirect recognition of PS and PS-associated moieties (90). However, the content of microparticles does not appear to be degraded in the phagolysosome. Microparticle constituents are often integrated within the phagocyte machinery and influences the cell function, differentiation, and activation state (161–168), indicating that internalized material is not routed to a conventional phagocytic route. Even synthetic particles induce NETs, and this effect is strictly dependent on their size (169). To the best of our knowledge, little is known on the possible action of microparticles on the ability of neutrophils to phagocytose particulate substrates.

## Mechanisms of the Choice

The size of the particulate dramatically influences the outcome of its interaction with neutrophils (170, 171). Neutrophils efficiently phagocytose several microorganisms. After internalization, neutrophil granules are rapidly mobilized via mechanisms dependent on small GTPases and on interacting proteins. Primary azurophilic granules, which contain preformed microbicidal moieties including myeloperoxidase and neutrophil elastase, eventually fuse with phagosomes (172). The phagocyte NADPH oxidase NOX2 complex assembles within the resulting phagolysosome, and electrons are transferred to molecular oxygen, with massive production of reactive oxygen species (ROS) into the lumen (173, 174). The presence of ROS combined with the neutrophil enzymes guarantees the killing of internalized microbes and the further degradation of the internalized particulate.

Myeloperoxidase, elastase, and ROS are in parallel key signals in the generation of NETs. In this case, signals that activate neutrophils lead to an oxidative burst and to the generation of ROS in the absence of a competent phagolysome. Thus, ROS cannot promote the focused release of neutrophil enzyme within the vesicle, contributing to dismantle the internalized cargo. ROS promote the release of the azurosome complex, which contains among other components myeloperoxidase and elastase, from the membrane of azurophilic granules into the cytosol. The complex then binds to and degrades the actin-based cytoskeleton. This event is a critical checkpoint.

(i) The degradation of the cytoskeleton is required to allow proteases to enter the nucleus (13, 18). Elastase and myeloperoxidase in particular concur to favor the decondensation of the chromatin *via* a pathway dependent on the ability of elastase to cleave histones but independent of the enzymatic activity of myeloperoxidase (17). Interference with the activity of elastase or complete absence of myeloperoxidase prevents the formation of NETs. When neutrophils have been previously engaged in phagocytosis, elastase and myeloperoxidase are sequestered within the phagolysome. They cannot reach the cytosol and eventually the nucleus and are not available for chromatin decondensation making it impossible for the phagocyte to generate NETs (18, 170).
(ii) Conversely, the integrity of the cytoskeleton is required to phagocytose particulate substrates. Neutrophils dismantle to generate NETs, the actin-based cytoskeleton, and are not competent anymore to phagocytose particulates. Small Rac GTPases, components of the NADPH oxidase NOX2 complex and required for the generation of NETs, regulate the cytoskeleton dynamics and adhesion (175, 176), providing a molecular link between the cytoskeleton remodeling and the requirement of neutrophil adhesion to solid substrates *in vitro* and *in vivo* for NETs generation (128).

NADPH oxidase activity is optimal at an intracellular pH of 7.5. An acute, transient drop in intracellular pH, dependent on H+ ions generated as a consequence of the NADPH oxidase activity, ensues the phagocytosis of opsonized bulky particulates (177) and acidic environments impair NET formation (178). Increased pH in contrast favors NET generation, possibly influencing the natural history of pancreatitis, where aggregated NETs occlude ducts and cause tissue injury (178, 179). Further studies are necessary to verify the effect of variation of the tightly regulated intracellular pH on the fate of neutrophils challenged with particulates.

Pathways leading to NET generation differ in terms of dependence on oxygen species, of kinetics of the process, and of the fate of the involved neutrophils, which can either die or survive after NET generation conserving at least some of their biological function (13, 180–182). Specifically MAPKs such as ERK and p38 regulate NOX2-dependent generation of NETs (183–185). The extent of activation of JNK/SAPK determines the response to synthetic and microbial stimuli, regulating the overall efficiency of ROS production and the ensuing NET generation (186). NET generation elicited by calcium ionophores relies on mitochondrial ROS and the calcium-activated small conductance potassium SK3 channel but is relatively independent of ERK activation (185, 187, 188). The existence of independent pathways leading to the generation of NETs endowed with potent biological action in the microenvironment that might compensate one for the other (13, 19, 182) supports the evolutionary importance of neutrophil activation. A ROS-independent, fast and vital pathway is apparently the first to be activated in neutrophils challenged with activated platelets and apoptotic cells. Depending on the environmental conditions, other outcomes can be envisaged.

The nucleus is not a passive target of the action of granular and cytosolic enzymes. At least two signals normally involved in chromatin architecture and function, DEK and HMGB1, actively regulate NET generation. Both molecules have a “double life.” Besides their action in the nucleus, they can be extruded or actively released in the extracellular environment where they regulate the inflammatory response. DEK, a highly conserved phosphoprotein involved in the control of genomic stability, is both an autoantigen and a chemoattractant signal whose role has been so far only partially characterized (189). Neutrophils release DEK into the extracellular space, and its presence is necessary for NETs generation, possibly because it stabilizes NET architecture in the extracellular space (16). To the best of our knowledge, little is known on the possible action of DEK in regulating neutrophil phagocytosis.

HMGB1 is as well a non-histone protein with an architectural function in the living cells [see above and (135, 190–192)]. Released in the extracellular environment, it represents the prototypic and so far the best characterized DAMP/alarmin signal (193–195). HMGB1 is a potent inducer of autophagy (196) and NETs generation (58, 131). HMGB1 released by activated platelets, activated leukocytes, and necrotic cells influences leukocyte functions, favoring neoangiogenesis (197), a tumor-permissive environment in experimental models (198, 199) and—possibly *via* NET induction—favoring a prothrombotic state in tumor-bearing patients (135). HMGB1 is also an effective inhibitor of phagocytosis (200–203). It is tempting to hypothesize that cytosolic, extracellular and nuclear HMGB1 can act in a coordinated manner, facilitating the survival and the adoption of the most effective response of a neutrophil challenged with a phagocytic substrate. Further studies are needed to test this hypothesis.

## A Metabolic Switch?

**Figure 2:**
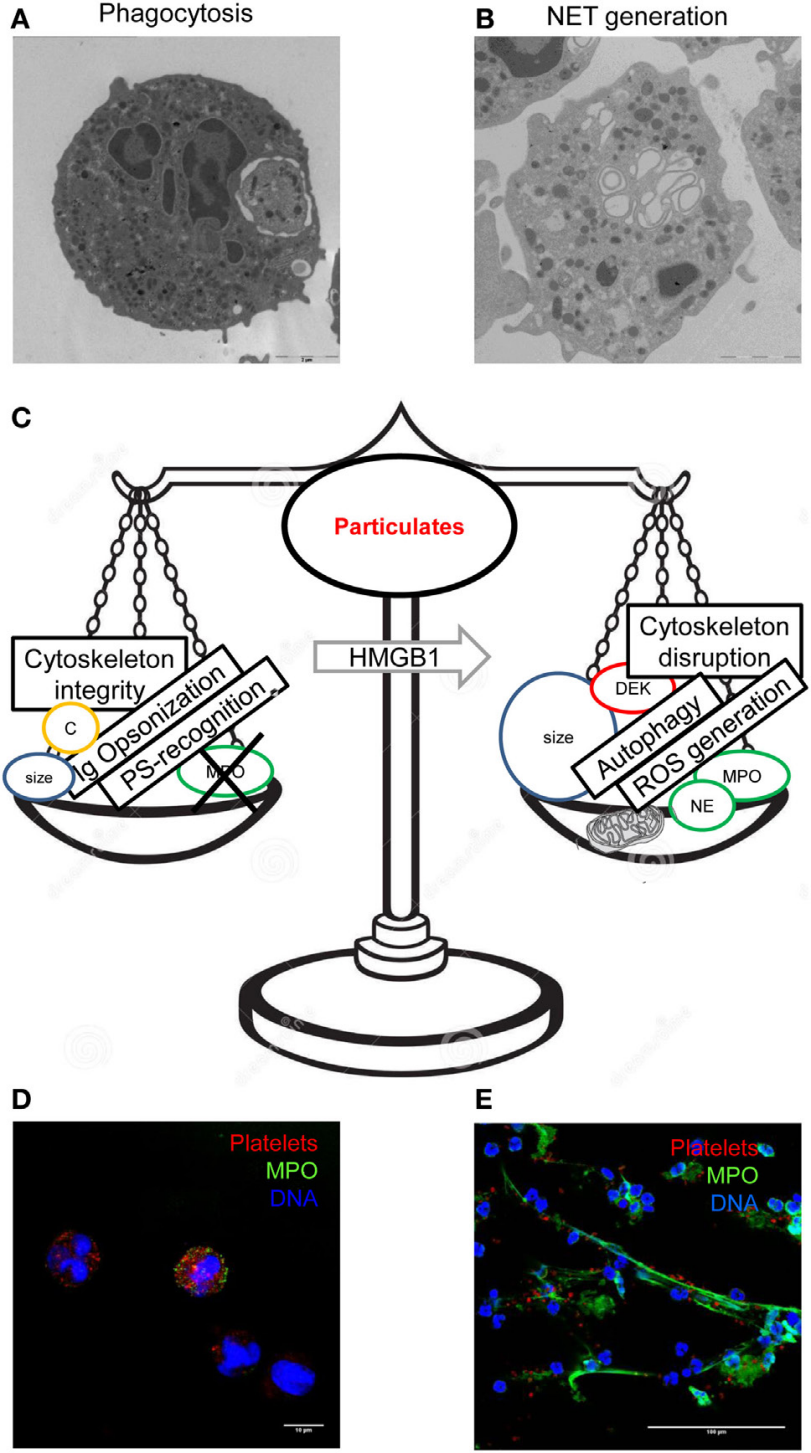
Integration of multiple signals leads to the final decision of the neutrophils challenged with a bulky particulate substrates, whether to phagocytose it or to generate neutrophil extracellular traps (NETs). Phagocytosed blood platelets were revealed by transmission electron microscopy in blood neutrophils of patients with *polycythemia vera* **(A)**. Neutrophils of healthy donors challenged with autologous activated platelets acquire a typical appearance at electron microscopy **(B)**. Several factors regulate phagocytosis and NET generation differentially, prompting one event to negatively regulate the other **(C)**. Internalization of red fluorescent platelets by neutrophils **(D)** and generation of extracellular threads of DNA (blue) decorated with myeloperoxidase (green color) was detected by confocal microscopy. **(A,D)** had originally been published in Ref. (68), **(B)** in Ref. (60), and **(E)** in Ref. (58).

Other mechanisms probably concur to explain the dichotomic nature of the neutrophil choice between phagocytosis and NET generation. Phagocytosis implies the sudden increase of the cell actual content depending on the ingestion of the phagocytic cargo with its own lipids, nucleic acids, proteins, etc. The metabolic pathways that allow the phagocyte to handle the further burden of internalized material have been only partially elucidated (204, 205). The fine regulation of the mitochondrial function appears of crucial importance, with the total mitochondrial membrane potential that directly impacts on the efficacy of the clearance *in vivo*, eventually leading to the termination of the apoptotic meal, i.e., to the failure of phagocytes that have internalized/are processing the prey to phagocytose further particulate substrates (206). A similar energetic constrain might restrict other potentially energy-consuming activities of the cell, such as initiation of the oxygen burst or the complete redistribution of the intracellular nuclear and granular content, which is a prerequisite of the release of NETs in the extracellular environment. Figure 2 depicts a schematic representation of events that might influence the decision of the neutrophil challenged with phagocytic substrates, whether to internalize and process it or to generate NETs. From the teleological point of view, to limit neutrophil reactivity after phagocytosis might restrict collateral damages caused by phagocytes persistently stimulated when their initial response has failed to clear the inflammatory *noxa*. Conversely, the systemic inflammatory response and enhanced thromboembolic risk, which are hallmarks of diseases associated with the failure of the phagocytic ability of neutrophils or macrophages, such as SLE, might thus reflect among other causes the lack of the calming effects of the phagocytic meal.

## Author Contributions

All authors listed have made substantial, direct, and intellectual contribution to the work and approved it for publication.

## Conflict of Interest Statement

The authors declare that the research was conducted in the absence of any commercial or financial relationships that could be construed as a potential conflict of interest.
